# Using GFP-Tagged *Escherichia coli* to Investigate the Persistence of Fecal Bacteria in Vegetated Wetlands: An Experimental Approach

**DOI:** 10.3390/antibiotics9060335

**Published:** 2020-06-18

**Authors:** Emilia Chiapponi, Charles P. Henriot, Xavier Bertrand, Didier Hocquet, Gudrun Bornette

**Affiliations:** 1 BIGEA–Biological, Geological and Environmental Sciences, Via S. Alberto 163, Ravenna Campus, University of Bologna, 40126 Bologna, Italy; 2 UMR 6249, Laboratoire Chrono-Environnement, CNRS-Université de Bourgogne Franche-Comté, 25030 Besançon, France; xavier.bertrand@univ-fcomte.fr (X.B.); didier.hocquet@univ-fcomte.fr (D.H.); gudrun.bornette@univ-fcomte.fr (G.B.); 3 Hygiène Hospitalière, Centre Hospitalier Universitaire de Besançon, 3 Boulevard A. Fleming, 25030 Besançon, France

**Keywords:** fecal bacteria, wetlands, macrophytes, antimicrobial compounds, *Escherichia coli*, GFP

## Abstract

The contamination of surface water by pathogenic bacteria of human origin is an important public health issue. Wetlands can be contaminated with fecal bacteria by water originating from different sources, such as wastewater treatment plants and agriculture. *Escherichia coli* is a commensal of the human gut flora and the major indication of fecal contamination in surface water. Little is known about the association between fecal bacteria and submerged macrophytes and how this may influence the water quality. We questioned whether macrophytes enhance or inhibit the bacterial growth in wetlands. For this purpose, we grew four different species of macrophytes (*Mentha aquatica*, *Baldellia ranunculoides*, *Sparganium emersum* and *Elodea canadensis*, in mono- or multispecies cultures) in aquatic rhizotrons and inoculated the devices with a fluorescent strain of *Escherichia coli* (producing a green fluorescent protein) to simulate the fecal contamination of wetlands. Bacterial survival was monitored by measuring the fluorescence for 19 days. We found (i) that contaminated sediments did not release *E. coli* in the water column in lentic conditions and (ii) that monocultures of *E. canadensis*, *M. aquatica* and *S. emersum* reduced the *E. coli* concentration in the water column. This suggests that aquatic plant species may be used in constructed wetlands to clear surface freshwater from bacteria of fecal origin.

## 1. Introduction

The pathogenic bacterial contamination of surface water is a major health risk for the human population and a threat to future water supplies [1,2]. New contemporary problems, such as antibiotic resistance, could aggravate the existing situation [3].

Fecal bacteria contaminating freshwaters originate from both punctual sources (e.g., wastewater treatment plants (WWTPs) [4], hospitals [5] and agriculture [6]) and nonpunctual ones (e.g., sewage sludge [7], manure [8], rainwater runoff [9] and percolation from fertilized fields [10]). All these sources contain pathogens that contribute to the fecal peril. Many transnational organizations (e.g., WHO and the European Union) set standards and guidelines for assessing the water quality and indicate *Escherichia coli* as a major indicator for fecal contamination [3]. This species is a commensal of human and animal guts and is released into the environment through feces [11,12]. Once in the environment, the bacteria disperse and reach different ecosystems such as surface water, including wetlands, in which they potentially survive [13,14].

Natural wetlands have beneficial effects on the water quality, while increasing the habitat diversity and maintaining ecological sustainability [15,16]. However, very little is known about the survival of fecal bacteria in these environments. On the one hand, oxygenation of the rhizosphere by the vegetation, crucial for the survival of microorganisms, may promote the survival of fecal bacteria in wetlands [17]. On the other hand, the shade and the allelopathic compounds excreted by the vegetation may negatively affect fecal bacteria in wetlands [18,19]. Several experimental studies have shown that constructed wetlands vegetated with macrophytes efficiently reduce the bacterial load, outlining their potential bioremediation properties [20,21]. The mechanisms of bacteria clearance in these environments may be numerous: natural die-off, UV radiations on the cell wall of bacteria, protozoan predation, antimicrobial substances secreted by macrophyte roots, etc. [22,23].

Here, we aimed at determining experimentally the fate of bacteria of fecal origin in vegetated wetlands and the remediation potential of macrophytes that naturally colonize these wetlands, using *E. coli* as a model. Several studies were carried out on constructed wetlands [22,24,25], whereas, to the best of our knowledge, only one has investigated the potential of natural wetlands [26]. The subject remains poorly studied, and to our knowledge, no study has been aimed at comparing experimentally the efficiency of several macrophyte species colonizing natural wetlands at controlling fecal bacteria survival and multiplication.

To do so, we grew different macrophytes in rhizotrons, which species were selected by the demonstrated or potential production of the secondary compounds and antimicrobial activity. We then inoculated the rhizotrons with GFP-tagged *E. coli* (GFP-*E. coli*) and followed bacterial fluorescence for 19 days. This ex-situ study was carried out implementing nondestructive, cost-effective and simple methods to assess the spatiotemporal dynamics of the bacterial contamination without compromising the plants and rhizotrons settings.

## 2. Results

The bacterial concentration was monitored for 19 days in mixed-species, single-species and unplanted rhizotrons inoculated with a fluorescent GFP-*E. coli* strain. In all inoculated rhizotrons, fluorescence—and so, the concentration of bacteria—increased until reaching a peak in the third day after inoculation. The bacterial concentration started to decrease drastically around day six, until reaching values under the limit of detection (LOD) at day eight.

### 2.1. Influence of the Type of Inoculation in Bacterial Survival

The limit of detection (LOD) was 4.91 × 10^5^ CTCF (corrected total cell fluorescence) for the mixed-species rhizotrons and 4.07 × 10^5^ CTCF for the unplanted ones. Only fluorescence from day one to day eight was detectable and, thus, considered.

**Figure 1:**
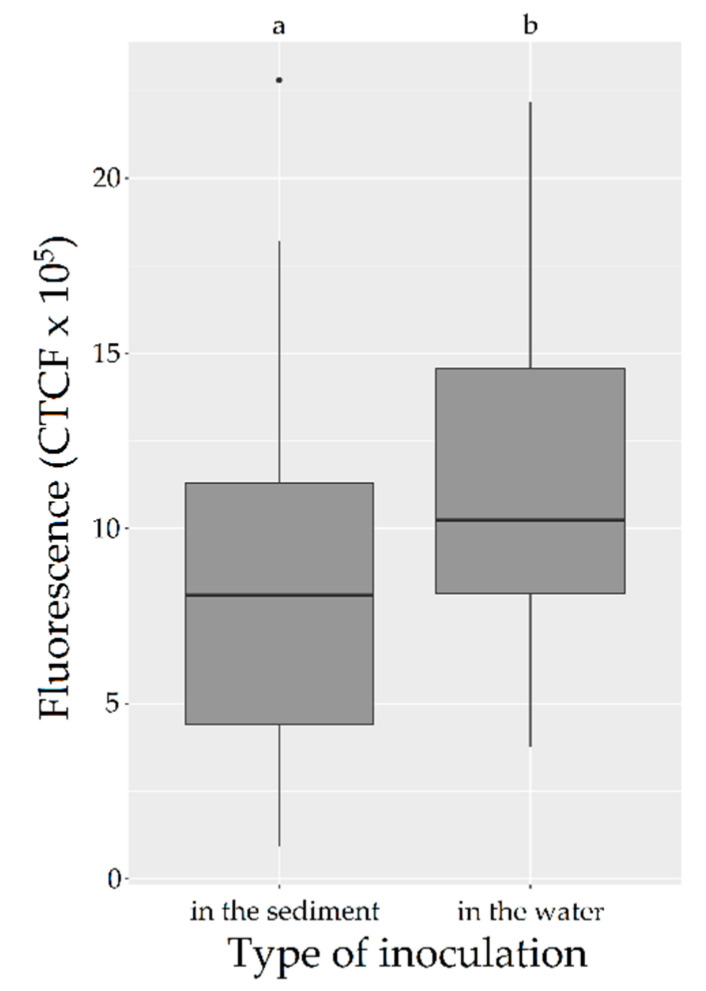
Effect of the type of inoculation—in water (*n* = 6) or in sediment (*n* = 6)—on the fluorescence of GFP-*Escherichia coli* in the rhizotrons. Boxplots of fluorescences measured on days 1, 2, 3, 6 and 8 (fluorescences > limit od detection (LOD)) in the water of rhizotrons inoculated with GFP-*E. coli*. Using an ANOVA with a multiple comparisons Tukey’s test on the l-mm, testing the effects of the type of inoculation on the fluorescence (i.e., bacterial concentration) of the water column. a and b are significantly different (*p*-value (ANOVA) < 0.01). CTCF: corrected total cell fluorescence.

The location of the bacterial inoculation (water or sediment) had an effect on the florescence measured in the rhizotron water (ANOVA on the l-mm, *p*-value = 0.0061). The rhizotrons that were inoculated in the sediment (*n* = 6; three with the mixture of four plants and three without plants; Table 1) showed less fluorescence than the ones inoculated in the water (*n* = 6; three with the mixture of four plants and three without plants; Table 1) (Figure 1, Tukey’s test on the l-mm). No interactive effect between the presence/absence of plants and the type of inoculation was observed.

### 2.2. The Role of Vegetation and Plant Species in Bacterial Survival

The LOD was 1.33 × 10^5^ CTCF for *Baldellia ranunculoides* rhizotrons, 1.57 × 10^5^ CTCF for *Elodea canadensis* rhizotrons, 1.89 × 10^5^ CTCF for *Mentha aquatica* rhizotrons and 1.07 × 10^5^ CTCF for *Sparganium emersum* rhizotrons. Only fluorescence from the day one to day eight was detectable and, thus, considered.

**Figure 2:**
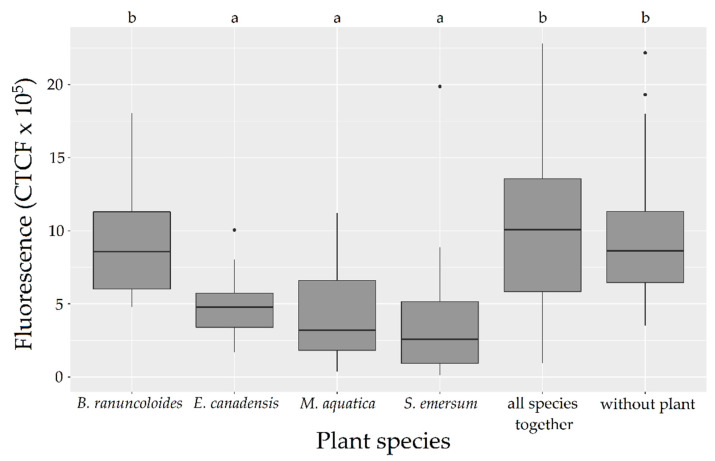
Effects of *Baldellia ranunculoides* (*n* = 3), *Elodea canadensis* (*n* = 3), *Mentha aquatica* (*n* = 3), *Sparganium emersum* (*n* = 3), the mixture of these four species together (*n* = 3) and the absence of plants (*n* = 3) on the fluorescence of GFP-*E. coli* in the rhizotrons. Boxplots of fluorescences measured on days 1, 2, 3, 6 and 8 (fluorescences > LOD) in the water of rhizotrons inoculated with GFP-*E. coli*. Using an ANOVA with a multiple comparisons Tukey’s test on the l-mm, testing the effects of plant species on the fluorescence (i.e., bacterial concentration) of the water column. a and b are significantly different (*p*-value (ANOVA) < 0.001). The dots are outliers.

The fluorescence did not differ between the rhizotrons without plants and those planted with the set of four species (ANOVA on the l-mm, *p*-value = 0.89). For the single-species vegetated rhizotrons (*n* = 12; four plant species in three replicates each; Table 1), the measured fluorescence differed between rhizotrons according to the species planted (ANOVA on the l-mm, *p*-value = 0.000024). The rhizotrons containing *E. canadensis, M. aquatica* or *S. emersum* showed less fluorescence than those containing *B. ranunculoides.* In the same way, the measured fluorescence differed between rhizotrons planted with *B. ranunculoides* (*n* = 3; Table 1), *E. canadensis* (*n* = 3; Table 1), *M. aquatica* (*n* = 3; Table 1), *S. emersum* (*n* = 3; Table 1), the mixture of these four species together (*n* = 3; Table 1) and the ones without plants (*n* = 3; Table 1) (ANOVA on the l-mm, *p*-value = 3.4 × 10^–10^). The rhizotrons containing *E. canadensis, M. aquatica* or *S. emersum* were less fluorescent than those containing *B. ranunculoides*, the four tested species together (mixed-species rhizotrons) and those containing no plants (Figure 2; Tukey’s test on the l-mm).

No difference of fluorescence was observed between the six mixed-vegetated rhizotrons according to their vegetation densities, as these were comparable (i.e., 50%, 50%, 50%, 75%, 75% and 25% in the six vegetated rhizotrons, measured the third day after inoculation).

We did not observe any fluorescent spot in the rhizosphere (mono- and poly-species rhizotrons) during the experiment.

## 3. Discussion

The aim of this study was to assess experimentally the fate of fecal bacteria in vegetated wetlands and their remediation potential according to the vegetation. To do so, we followed the fluorescence of a GFP-*E. coli* in an experimental model.

In all rhizotrons, we observed an increase of fluorescence in the first days following inoculation, maybe because the bacteria were inoculated with their growth broth. Between days three and eight, even if *E. coli* is mobile, we can assume the sedimentation of the bacteria, since there was no flow and since the measured fluorescence increased in the lower part of the rhizotrons [27,28]. Fluorescence was under the LOD from day eight. We first hypothesized that the bacteria lost the plasmid that borne the GFP-encoding genes, as already reported [29]. To test this possibility, we sampled water aliquots from each rhizotron at day eight and streaked them on a growing medium (Mueller-Hinton agar) further incubated overnight at 37 °C. Both fluorescent and nonfluorescent *E. coli* grew, but the fluorescent *E. coli* largely dominated (species identification checked with a MALDI-TOF MS). The decay of fluorescence thus rather indicated that most GFP-*E. coli* did not survive in the rhizotrons after day eight. The specific conditions of the rhizotrons (i.e., no flow, no oxygenation of the sediment, no nutrient income and use of tap water) probably accounted for the poor survival of GFP-*E. coli*.

This study aimed to test the capability of natural wetlands to remove bacterial contaminations through vegetation, depending on the source of contamination (water column or sediment). Concerning the source of contamination, the bacterial fluorescence was lower in the water column of rhizotrons inoculated in sediments compared to those inoculated in the water. On the one hand, bacteria found nutrients and protection against predators in the sediment [30]. On the other hand, some studies observed a rapid decay of bacteria in the sediment due to the lack of dissolved oxygen and the absence of water column mixing, as it may be the case in the present experiment [29]. A substantial oxygen concentration in the sediment and water is crucial for a sufficient and visible (in terms of brightness) GFP-gene expression of the transformed plasmid (to allow visual identification within a fine sediments matrix [29]. Thus, the lentic condition of this experiment resulted firstly in a decay of the detected fluorescence and, later, (possibly due to a lack of nutrients) in bacterial population numbers [31]. As the nondestructive method used in this work did not allow to monitor the survival of bacteria in the sediment, it is not possible to conclude on the bacteria survival in this compartment. The present results suggest that the fecal bacterial load, for the most part, was not transferred from the sediment to the water column, either because of habitat preference or poor survival. This suggests that, under lentic conditions, the fecal contamination of the sediment may not significantly contribute to the contamination of the water column in natural wetlands [32]. Moreover, no fluorescent spot was observed near the roots on the inclined side of the planted rhizotrons. This could suggest either that bacteria did not survived in the sediment [33] or that the rhizosphere is not a favorable habitat for fecal bacteria, contrary to what other studies suggested [34].

Concerning the effect of vegetation, we found that monocultures of *E. canadensis, M. aquatica* and *S. emersum* reduced the survival of GFP-*E. coli*, whereas monocultures of *B. ranunculoides* and four-species cultures had no effect (Figure 2). *E. canadensis, M. aquatica* and *S. emersum* produced secondary compounds [35,36,37]. In contrast, secondary compounds of *B. ranunculoides* are poorly known [38,39]. The acetone extract from *S. emersum* (made of flavonoids, essential oils, phenylpropanoid glycosides and aromatic alkenes) has a high antimicrobial effect, which may account for the observed decay of bacteria [36]. Previous observations already indicated a negative relationship between *Elodea* and algae or cyanobacteria, and this species has been reported to have a medium allelopathic activity [40]. *Elodea* species contain diglucuronides of the flavones luteolin, apigenin and chrysoeriol and a yet-unidentified phenolic acid similar to caffeic acid [41], and various flavonoids have been demonstrated to inhibit the growth of Gram-negative bacteria [42]. *Mentha aquatica* benefits from a wider knowledge on its role, being largely used in constructed wetlands [43]. While analyzing its essential oils, a total of 34 compounds have been identified. Menthofuran (70.5%) was characterized as the main component, with limonene and pmenthone constituting 9.42% and 7.20% of the oil [44]. The results of the current research revealed that the essential oils exhibited moderate antibacterial effects against the microorganisms—in particular, against Gram-positive bacteria [45,46]. On the other hand, not much is known about *B. ranunculoides*, and the study of its biochemistry diversity is still in its early stages. All *Baldellia* spp. have very strong coriander-like smells whose chemical composition has not yet been studied [47]. It is therefore unclear, any chemical affinities with the aldehydic smell of *Coriandrum sativum* (*Apiaceae*). On the other hand, species of *Alisma* (closely related to *Baldellia* spp.) are reported to produce phenolic glycosides with caffeic acid, chlorogenic acid and quercetin [48]. The content of *B. ranunculoides* extracts are yet to be identified and tested on microbial activity. Even if the observed reduction in the bacterial concentration was rather low compared to conventional remediation processes used in water treatments, for example, the results suggest that *E. canadensis*, *M. aquatica* and *S. emersum* could be used in constructed wetlands for their capacity to limit bacterial survival and/or multiplication. However, more information is still needed for identifying the identity and quantity of allelopathically active compounds involved and on the factors influencing their production and release by macrophytes. Several studies suggest that the allelopathic effect is also very specific and deeply connected to the macrophyte species and its characteristics [33]. Indeed, plant exudates have the potential to act directly either on resources, competition or chemical interferences between plants and play a direct role in allelopathy mediation [36]. Furthermore, the effects of these secondary compounds on the rhizosphere, hosting bacterial communities essential to the development of vegetation, need to be better clarified.

These results must be nuanced, because multispecies and unvegetated rhizotrons did not show a significant difference in terms of bacterial survival. There is still no clear evidence that, by combining different species with known effects, it would result in a better treatment efficiency for a single contaminant compared to a treatment implying a monoculture [49]. As of now, there is no consistent accordance favoring a monoculture, despite mixed wetlands. Indeed, some studies have indicated that species competition may affect the nutrient removal and vegetation stability in constructed wetlands [50]. Mixed wetlands can be more or less efficient than monocultures, depending on the plant species (growth speed, biomass, competition ability and stubble growth attributes) used in both wetlands and on whether the mixed community can maintain a relatively stable state [51].

It is also possible that the concentration of a given exudate may need to reach a certain value to be effective. This could have implied that the concentrations of the secondary compounds did not reach a breakthrough concentration in the mixed rhizotrons. Monospecies environments may be more efficient, because the allelopathic compound would be much more abundant.

The noninvasive method used in this experiment allowed monitoring the bacterial concentration in the water column of the different rhizotrons for 19 days, without using the classical microbiology technics (more expensive, longer, laborious and potentially invasive). Nevertheless, on the one hand, this method has made it impossible to monitor the bacterial concentration in the sediment. This issue needs to be further explored to conclude on the potential role of the macrophyte rhizosphere as a reservoir of fecal bacteria. On the second hand, the method used in this work did not make it possible to investigate the potential shelter role of plants for bacteria (fixation and formation of biofilm on the surface of submerged parts and presence in vegetal tissues), as presented in several studies. Finally, while this experimental study allowed a better understanding on the fate of *E. coli* in vegetated wetlands, interactions between fecal bacteria and the natural bacterial community present in this habitat should be further investigated for a deeper perspective of fecal bacteria fate in natural wetlands.

It is desirable, in view of their relevance in bioremediation, to focus future studies not only on separate plant species as isolated individuals but, also, on considering the effects of different species as an interconnected whole and the complexity of their allelopathic effects. This knowledge is important for understanding the present existing mechanisms in natural wetlands that can drive the survival or removal of fecal bacterial populations and pollutants’ fate and to face the environmental bacterial contamination with effective remediation strategies.

## 4. Materials and Methods

### 4.1. Ecosystem and Conditions of Reference

To ensure that the experimental conditions were as close as possible to the natural ones (sediment composition, plants and temperature), we referred to natural wetlands of the Ain River in the Jura Massif (France) that have already been studied for bacterial contamination [14]. Rhizotrons were filled with artificial sediment prepared according to the sediment composition of six natural wetlands of the Ain River and those referred in the literature [52,53]. Hence, the sediments used were composed by 70% silty sand (supplied by a local quarry from the Ain River floodplain), 15% clay (kaolinite) and 15% organic matter (190 g.m-3 N, 100 g.m-3 P2O5 and 60 g.m-3 K2O). The mixture was poured in each rhizotron until covering 10 cm of the box height. Then, 2.5 L of tap water was gently poured, and the rhizotrons were left to settle for 48 h before plant transplant.

Four macrophyte species have been transplanted in the rhizotrons. Three species were chosen based on their abundance in natural wetlands and their referred potential to affect the bacterial community or to product the active secondary metabolites: *E. canadensis* [40], *M. aquatica* [43] and *S. emersum* [37]. The fourth species was *B. ranunculoides* because of its abundance in natural wetlands and to test its unknown effects on fecal bacteria [39,40]. The plants were collected in wetlands of the Ain River (*M. aquatica*, 46°00′36.3″ N 5°17′59.3″ E; *B. ranunculoides*, 45°58′51.1″ N 5°16′57.4″ E and *S. emersum* and *E. canadensis*, 45°58′04.7″ N 5°17′37.7″ E). Plants were stored at 5–8 °C for the time to transport them from the field to the laboratory (5 h), washed and transferred into the rhizotrons. The experiment room was kept at 18 °C to reflect the water temperature of natural wetlands.

### 4.2. Experiment Set up

#### 4.2.1. Rhizotrons

Thirty rhizotrons (polystyrene boxes of 27 cm H × 20 cm L × 10 cm W) were filled by 10-cm height of sediment. For comparing the effect of vegetation versus no vegetation on the bacterial success, we planted the four macrophyte species together (one individual per species) in seven rhizotrons: three were inoculated with GFP-*E. coli* in the water, three were inoculated with GFP-*E. coli* in the sediment and one served as a control (mixed-species vegetated but not inoculated with GFP-*E. coli*) (Table 1). We also prepared 7 rhizotrons without vegetation: three were inoculated with GFP-*E. coli* in the water, three were inoculated with GFP-*E. coli* in the sediment and one served as a control (not vegetated and not inoculated) (Table 1). Finally, for studying the effect of a given species on the bacterial success, 16 rhizotrons were planted with four individuals of each of the four different species of macrophytes (four rhizotrons per species—among which, one was not inoculated and served as a control) (Table 1). We only tested the effects of the inoculation (water versus sediment) for mixed-species and unvegetated rhizotrons (Table 1). The rhizotrons were inclined at 30° to observe the possible driving effects of the rhizosphere on the bacterial community [54,55]. The external part of the rhizotrons corresponding to the sediment layer was covered with black plastic film to reproduce natural dark conditions that prevail in natural sediment. We applied a 12-h photoperiod with a growth lamp with blue led light at 30 W (Digyssal, Guangzhou Hua Dun Electronic Technology Co. Ltd., Guangdong, China).

In order to verify that plant size did not differ significantly between replicates, and to assess the possible effect of plant size on bacterial survival, we measured the density of vegetation at the beginning of the experiment and three days after inoculation using 5 classes of density (0%, 25%, 50%, 75% and 100%).

#### 4.2.2. Inoculation with GFP-*E. coli*

We used a fluorescent and nonpathogenic strain *E. coli* GFP ATCC^®^ 25922GFP™ (American Type Culture Collection, Manassas, VA, USA). It harbors a multicopy vector encoding the green fluorescent protein GFPmut3. The GFPmut3 fluorophore has an excitation maximum at 501 nm and an emission maximum at 511 nm, compatible with usual fluorescence detection technologies. The plasmid-borne bla gene confers the resistance to 100 µg/mL of ampicillin. This *E. coli* strain has been grown in a Luria-Bertrani (LB) broth overnight at 37 °C with gentle shaking to reach the growth plateau (~109 CFU/mL). Then, an aliquot of the culture of 6 mL with a concentration of 2.4 × 10^6^ CFU/mL was inoculated in rhizotrons via a sterile pipette for water inoculation and via sterile syringe connected to a tube to reach a 5-cm depth in sediments to simulate contaminations from the surface water and groundwater, respectively. To make the strain visible, it was excited at each measure by lightening the rhizotrons with a FastGene^®^ Blue/Green LED Flashlight (Nippon Genetics Europe GmbH, Dueren, Germany) emitting at 480–530 nm.

### 4.3. Image Analysis

#### 4.3.1. Camera and Settings

**Figure 3:**
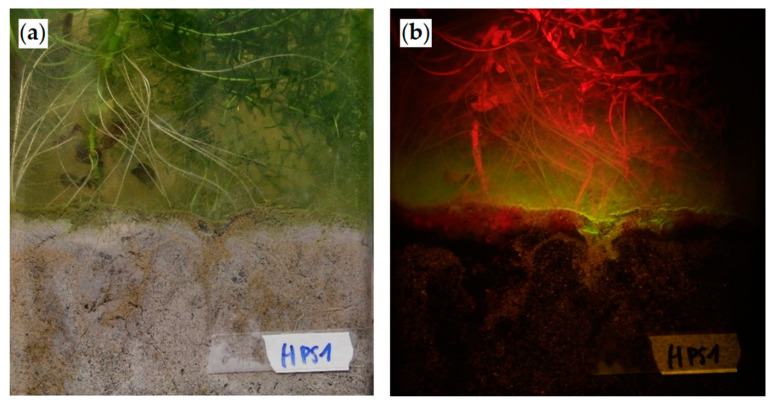
Example of planted rhizotrons after the inoculation of fluorescent *E. coli* (3rd day after inoculation). (**a**) Rhizotron under natural light. (**b**) Rhizotron under fluorescence conditions (enlightened only with a 480–530-nm light and observed with an amber filter). Photo obtained with an exposition of 13 s f/18 and ISO 800 with a Canon EOS 7D.

Unmodified digital single-lens reflex (DSLR) camera can be a successful tool for detecting fluorescence while being cost-effective, applicable to a wide range of studies and easy to use for the operators [56,57]. A DSLR camera Canon model Eos 7D (Canon Europe N.V., Amstelveen, The Netherlands) on a tripod with an amber filter on the front was used. A black cardboard was placed behind the photographed rhizotron in order to avoid reflection from the background. Each day of measurement, the rhizotrons were photographed in light and total dark conditions while enlightening them with green UV flashlight to spot fluorescence (Figure 3). To ensure the same lighting conditions, the camera and the lamp were kept each time at the same distance from the photographed rhizotron. Camera settings were kept the same throughout the whole duration of the experiment (1/50 s f/4 29 mm ISO 320 for light conditions and 13 s f/18 41 mm and ISO 800 for detecting fluorescence). Photos were taken on days 1, 2, 3, 6, 8, 10, 14 and 19 after inoculation. The limit of detection (LOD) was calculated for each different condition measuring the fluorescence in the control rhizotrons. Fluorescence readings lower than the LOD were removed from the analysis.

#### 4.3.2. Image Analysis

The images capturing fluorescence were treated with ImageJ2 ™ (National Institute of Health, Bethesda, MD, USA). This has been shown to be a useful tool for fluorescence intensity analysis [58,59]. The pictures were first converted as an 8-bit grayscale image. The colors channels were split in order to keep only the green fluorescence emitted from bacteria, reducing the noise from the red fluorescence emitted by plants and algae present in the rhizotrons. Noise from burned pixels in the image was also corrected and treated. Then, the CTCF was calculated by selecting three measurement areas (triplicates) with a mean of 20,380.3 square pixels in the water-sediment interface level. We then took three measurements of a mean area of 7318.6 square pixels of the nearby background to correct the fluorescence and calculate the CTCF as follows [60,61]:

*CTCF = Integrated density − (area of selected fluorescence × means fluorescence of the backgrounds)*

#### 4.3.3. Calibration Curve

**Figure 4:**
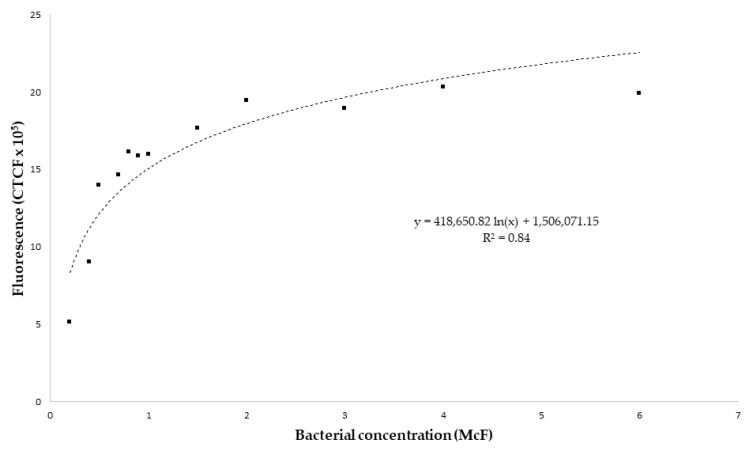
Fitting curve between the fluorescence measurements (CTCF) and the bacterial concentration (McF). Twelve vials with known concentrations of GFP-*E. coli* (0.2, 0.4, 0.5, 0.7, 0.8, 0.9, 1, 1.5, 2, 3, 4 and 6 McF) were photographed three times under the same conditions as the rhizotrons. The values presented are the means of these three measurements for each vial.

Increasing the concentration of GFP-*E. coli* was prepared in different vials (0.2, 0.4, 0.5, 0.7, 0.8, 0.9, 1, 1.5, 2, 3, 4 and 6 McF) and photographed them three times under the same conditions as the rhizotrons. The CTCF for each vial was calculated as described above, and we deducted a calibration curve between the retrieved CTCF and GFP-*E. coli* concentration (Figure 4). We obtained a curve of 12 points and with a R^2^ of 0.84 (Figure 4). The lowest concentration measured was 0.25 McF, which yielded 5.12 × 10^5^ CTCF, and the highest value measured was for 4 McF, even though it was not the highest bacterial concentration (6 McF), which yielded at 2.03 × 10^6^ CTCF.

### 4.4. Statistics

The normality and homoscedasticity of the data were tested using the Shapiro-Wilk test and Bartlett’s test, respectively. The dataset was square-root (sqrt)-transformed for reaching normality.

Four linear mixed-effects models (lmm) were performed, putting a random effect on the day of sampling to avoid a time-dependent artefact. The first lmm tested the effect of the type of inoculation (water versus sediment) on the fluorescence measured in the rhizotrons. The second lmm tested the effect of the four species together (mixed-species rhizotrons) against the absence of plants on the fluorescence measured in the rhizotrons. The third lmm tested the effect of the four plant species tested individually (single-species rhizotrons) on the fluorescence measured in the rhizotrons. An overall lmm, including the four single-species treatments, the mixed-species treatment and the unplanted rhizotrons were also assessed. An ANOVA was then performed on each lmm, followed by a Tukey’s test. The α value was set to 0.05. All analyses were performed with R 3.6.1 software [62].

## 5. Conclusions

In conclusion, the experiment demonstrated that aquatic plant species may play a role in the survival of bacteria of fecal origin that contaminates surface freshwater. In the lentic conditions of our experiments, we found (i) that contaminated sediments did not release *E. coli* in the water column in lentic conditions and (ii) that monocultures of *E. canadensis, M. aquatica* and *S. emersum* reduced the *E. coli* concentration in the water column.

## Figures and Tables

**Table 1:** Experimental design. Planted and unplanted rhizotrons inoculated with GFP-*Escherichia coli* in water or sediment.

Rhizotron Type	Plant Species	Type of Inoculation with GFP-*E. coli*		
		Water	Sediment	Control ^1^
Rizotrons planted with the four species together (*n* = 7)	*Baldellia ranunculoides, Elodea. canadensis,* *Mentha aquatica,* *Sparganiumemersum* together	*n* = 3	*n* = 3	*n* = 1
Rhizotrons without vegetation (*n* = 7)	Ø ^2^	*n* = 3	*n* = 3	*n* = 1
Rhizotrons planted with one single plant species (*n* = 16)	*Baldellia ranunculoides*	*n* = 3	Ø ^3^	*n* = 1
	*Elodea canadensis*	*n* = 3	Ø ^3^	*n* = 1
	*Mentha aquatica*	*n* = 3	Ø ^3^	*n* = 1
	*Sparganium emersum*	*n* = 3	Ø ^3^	*n* = 1

^1^ no bacteria inoculation. ^2^ no plants in rhizotrons without vegetation. ^3^ not tested.
